# Reference-based cell type matching of in situ image-based spatial transcriptomics data on primary visual cortex of mouse brain

**DOI:** 10.1038/s41598-023-36638-8

**Published:** 2023-06-13

**Authors:** Yun Zhang, Jeremy A. Miller, Jeongbin Park, Boudewijn P. Lelieveldt, Brian Long, Tamim Abdelaal, Brian D. Aevermann, Tommaso Biancalani, Charles Comiter, Oleh Dzyubachyk, Jeroen Eggermont, Christoffer Mattsson Langseth, Viktor Petukhov, Gabriele Scalia, Eeshit Dhaval Vaishnav, Yilin Zhao, Ed S. Lein, Richard H. Scheuermann

**Affiliations:** 1 J. Craig Venter Institute, La Jolla, CA USA; 2 Allen Institute for Brain Science, Seattle, WA USA; 3 School of Biomedical Convergence Engineering, Pusan National University, Busan, Korea; 4 LKEB, Department of Radiology, Leiden University Medical Center, Leiden, The Netherlands; 5 Pattern Recognition and Bioinformatics Group, Delft University of Technology, Delft, The Netherlands; 6 Broad Institute of MIT and Harvard, Cambridge, MA USA; 7 Science for Life Laboratory, Department of Biochemistry and Biophysics, Stockholm University, Stockholm, Sweden; 8 Biotech Research and Innovation Centre, Faculty of Health and Medical Sciences, University of Copenhagen, Copenhagen, Denmark; 9 Department of Biomedical Informatics, Harvard Medical School, Boston, MA USA; 10 Department of Pathology, University of California, San Diego, CA USA; 11 Division of Vaccine Discovery, La Jolla Institute for Immunology, La Jolla, CA USA; 12 Present Address: Chan Zuckerberg Initiative, Redwood City, CA USA; 13 Present Address: Genentech, South San Francisco, CA USA

**Keywords:** Cellular neuroscience, Neuroscience, Data integration

## Abstract

With the advent of multiplex fluorescence in situ hybridization (FISH) and in situ RNA sequencing technologies, spatial transcriptomics analysis is advancing rapidly, providing spatial location and gene expression information about cells in tissue sections at single cell resolution. Cell type classification of these spatially-resolved cells can be inferred by matching the spatial transcriptomics data to reference atlases derived from single cell RNA-sequencing (scRNA-seq) in which cell types are defined by differences in their gene expression profiles. However, robust cell type matching of the spatially-resolved cells to reference scRNA-seq atlases is challenging due to the intrinsic differences in resolution between the spatial and scRNA-seq data. In this study, we systematically evaluated six computational algorithms for cell type matching across four image-based spatial transcriptomics experimental protocols (MERFISH, smFISH, BaristaSeq, and ExSeq) conducted on the same mouse primary visual cortex (VISp) brain region. We find that many cells are assigned as the same type by multiple cell type matching algorithms and are present in spatial patterns previously reported from scRNA-seq studies in VISp. Furthermore, by combining the results of individual matching strategies into consensus cell type assignments, we see even greater alignment with biological expectations. We present two ensemble meta-analysis strategies used in this study and share the consensus cell type matching results in the Cytosplore Viewer (https://viewer.cytosplore.org) for interactive visualization and data exploration. The consensus matching can also guide spatial data analysis using SSAM, allowing segmentation-free cell type assignment.

## Introduction

Characterizing the spatial distributions of molecularly defined cell types is a shared goal of the Human Cell Atlas (HCA)^1^, NIH BRAIN Initiative Cell Census Network (BICCN)^2^, Human BioMolecular Atlas Program (HuBMAP)^3^ and related collaborative efforts. The core elements in this task include transcriptional classification and spatial localization of cell types, which involves integration of single cell and spatially-resolved transcriptomics to define and spatially match cell types through the analysis of combinatorial gene expression patterns in tissue sections. Single cell and single nucleus RNA sequencing (scRNA-seq) has rapidly progressed into a high throughput standardized methodology and has been used by many labs as a major workhorse for cell type classification in many organs. In contrast, spatial transcriptomics methods are still evolving, varying substantially in methodology, degree of multiplexing, cost, and throughput, and lacking consensus data standards and analysis methods.

Characterizing spatially-resolved cell types is essential in the brain in order to study the exceptional cellular heterogeneity and functional significance of its spatial organization. ScRNA-seq has revealed an unprecedented granularity of neuronal cell types in mouse and human brains^4–7^, providing a comprehensive landscape of cell type heterogeneity defined by their transcriptional profiles. Recently, a number of multiplex fluorescence in situ hybridization (mFISH) and in situ RNA sequencing methods^8–18^ have been reported for conducting spatial transcriptomics experiments at the cellular level. Each method is independently optimized for marker gene panel design, tissue processing, transcript sequencing, and imaging steps of the pipeline, requiring different strategies for data processing, quality control, and downstream analysis. The SpaceTx Consortium, an organized effort consisting of both experimental and computational working groups, took the lead to evaluate the performance of currently available spatially-resolved transcriptomics methods in high quality cortical brain samples, with the goal of building consensus maps of cortical cell type distributions based on combined analysis of single cell and spatially-resolved transcriptomics. The overarching effort of the SpaceTx Consortium will be described in a separate publication^19^.

One aim of the SpaceTx Consortium was to make probabilistic assignments of cell types and map their spatial distributions. Here we describe the quantitative meta-analysis of spatial transcriptomics data, with a focus on assigning spatial cell types using the reference cell types from scRNA-seq. In this manuscript, in situ image-based spatial transcriptomics data are analyzed and compared across spatial and computational methods for cell type determination on the same tissue. We present the results of these analysis efforts along with strategies for visualization of spatial transcriptomics data. Four spatial datasets from the SpaceTx Consortium and six computational methods are systematically evaluated in the following sections. Available datasets and reproducible work covered in this manuscript are publicly available at the SpaceTx website (https://spacetx.github.io/).

## Results

### Analysis overview

**Figure 1:**
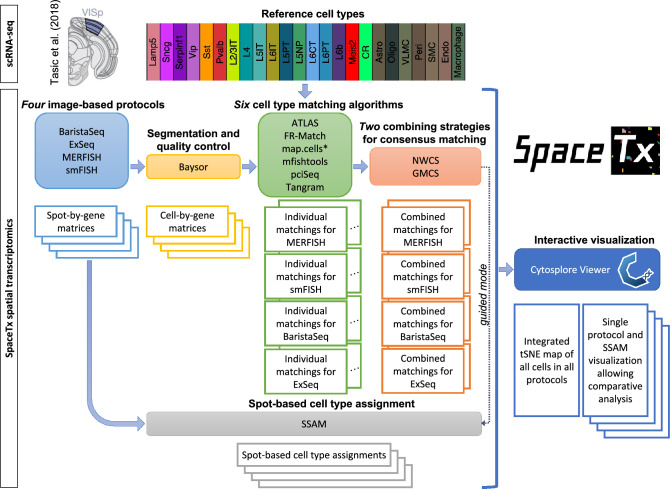
Overview of the SpaceTx analysis workflow. The reference scRNA-seq cell types of the primary visual cortex (VISp) of mouse brain are from Tasic et al.^4^. Spatial transcriptomics data were generated by four image-based experimental protocols (MERFISH, smFISH, BaristaSeq, and ExSeq). Segmentation and quality control were performed using a common procedure (Baysor). Six computational algorithms (ATLAS, FR-Match, map.cells*, mfishtools, pciSeq, and Tangram) for cell type matching were applied. Two meta-analysis strategies were used to combine the individual matching results. Spot-based segmentation-free cell type assignment was conducted using SSAM. All data and matching results can be viewed in Cytosplore Viewer (https://viewer.cytosplore.org).

This manuscript reports the collective efforts from the teams that participated in the SpaceJam Hackathon (https://spacetx.github.io/spacejam.html) organized by the SpaceTx Consortium. We explored multiple approaches to assign the spatial data with reference scRNA-seq cell type annotations and developed meta-analysis strategies to combine the cell type assignment results from multiple methods to reach consensus assignments (Fig. 1). We evaluated datasets from four image-based spatial methods (MERFISH^20,21^, smFISH (Allen protocol used in this study https://www.protocols.io/view/single-molecule-fish-x54v987q1l3e/v1), BaristaSeq^22^, and ExSeq^23,24^) applied to tissue section from the mouse primary visual cortex brain region (VISp)^4^. All mRNA detection data (spot-by-gene matrices) were segmented using the same segmentation procedure—Baysor^25^, which also included consistent quality control approaches for doublet and low-quality cell removal. The segmentation step produced the cell-by-gene matrices that were used to assign the spatially-resolved cell types to scRNA-seq reference cell types using cell type matching algorithms.

Teams of the SpaceTx Consortium explored six computational algorithms (ATLAS^26^, FR-Match^27,28^, map.cells*^4^, mfishtools^29^, pciSeq^30^, and Tangram^31^), which produced individual cell type assignments with various probabilistic assignment scores. To arrive at consensus cell type assignments, two meta-analysis strategies were developed to combine the individual assignments more quantitatively (Geometric Mean Combining Strategy, hereinafter GMCS), or more qualitatively (Negative Weighting Combining Strategy, hereinafter NWCS) (see “Methods” section). In parallel, spot-based cell type assignment was performed by SSAM^32^ using a guided mode, which partially borrows information from the combined assignment results. All spatial data and cell type assignment results were loaded into the Cytosplore Viewer (https://viewer.cytosplore.org) for interactive visualization and data exploration, where an integrated tSNE^33^ map for all annotated cells in all spatial methods are presented together with single method viewers for comparative analysis.

### In situ hybridization spatial transcriptomics data

The overarching SpaceTx Consortium project evaluated spatial transcriptomics technologies including multiple imaging-based spatial transcriptomics protocols alongside spatial sequencing methods, 10 × Visium, and Slide-seq. For the scope of this meta-analysis of computational evaluations, we focus on the in situ imaging-based protocols with data that has passed preliminary quality control. All data used and presented in this manuscript were generated by the experimental working group of the SpaceTx Consortium. Experimental details, including gene panel selection, tissue distribution and processing, image processing, etc., are available in^19^. Downloadable datasets used in this manuscript are publicly available at https://spacetx.github.io/data.html.

### Reference cell type taxonomy

The goal of this study was to produce an initial cell type matching of the spatially-resolved transcriptomics data to open access reference scRNA-seq cell type datasets (a.k.a. scRNA-seq-reference-based cell type assignment of spatial transcriptomics data). The reference mouse visual cortex (VISp) scRNA-seq data were reported in^4^, consisting of 14,249 cells with 116 cell types defined for VISp and anterior lateral motor cortex (ALM); the subset of cells collected from VISp were used in this study. With a focus on spatial gradients, the SpaceTx Consortium re-clustered the data to arrive at a reference cell type taxonomy that contains 191 consensus higher-resolution cell types at the most granular level and 24 cell type subclasses at the intermediate level (re-clustering details are in^19^). With fewer cells per study, fewer reads per cell, and limited probe sets, the granularity of spatial data collected in this project is not comparable to these most granular scRNA-seq cell types. Therefore, in this study we assigned the spatial data to the 24 cell type subclasses in the reference cell type taxonomy, which distinguishes major GABAergic, glutamatergic, and glial cell subtypes with layer-specific laminar patterning (a list of the reference cell type subclasses can be found in Fig. 1). General guidelines for selecting reference taxonomies for this study and others are provided in the “Discussion” section.

### Experimental protocols

As part of the SpaceTx Consortium, tissue sections were successfully collected from mouse VISp and evaluated using several image-based experimental methods, including MERFISH^20,21^, smFISH (Allen protocol used in this study https://www.protocols.io/view/single-molecule-fish-x54v987q1l3e/v1), BaristaSeq^22^, and ExSeq^23,24^ protocols. In general, the imaging-based protocols conduct multiple rounds of chemistry and microscopy to measure the location of individual mRNA molecules in the tissue section. Since each spatial method has unique requirements for numbers of genes and expression levels, each experimental protocol assembled different probe panels with specific gene sets in their design (Supplementary Table S1). Sensitivities of detection across protocols are reported in Table 1 in the SpaceTx Consortium paper^19^. The primary output from these imaging protocols is a spot-by-gene matrix, quantifying detected mRNA locations decoded from the image data.

**Table 1:** Summary of segmented and filtered data for each experimental protocol.

Protocol	Quality control filter	# Cells (before/after filtering and annotation)	# Genes
MERFISH	n_transcripts ≥ 50, elongation < 9, 2000 ≤ area < 45,000, avg_confidence > 0.8	6130/2150	258
smFISH	n_transcripts ≥ 50, elongation < 8, 500 ≤ area < 40,000, avg_confidence > 0.95	4841/2360	22
BaristaSeq	n_transcripts ≥ 20, elongation < 8, 10 ≤ area < 300, avg_confidence > 0.95	14,095/4432	79
ExSeq	n_transcripts ≥ 20, elongation < 10, 5000 ≤ area < 1,500,000, avg_confidence > 0.8	1504/1271	42

The filtered cell-by-gene matrices are available at https://spacetx.github.io/data.html.

### Segmentation and quality control

The segmentation step produces a cell-by-gene expression matrix from the spot-by-gene matrix for downstream analysis. For this purpose, the Baysor algorithm was used because it has been reported to outperform other segmentation tools in terms of yielding better segmentation accuracy, increased number of cells detected, and improved molecular resolution by considering joint likelihood of transcriptional composition and cell morphology^25^. Baysor was applied for cell segmentation across all imaging-based protocols to achieve comparable quantifications from the different protocols by using the same segmentation method. Low quality cells were filtered based on the Baysor cell segmentation statistics, e.g., number of transcripts per cell, elongation characteristics, cell area values, and average confidence scores of segmentation. Cells not passing the quality control filter and cells located outside of VISp excluded from further analysis. The boundaries of VISp were based on cell density (marking the end of layer 6b and beginning of white matter) and visual comparison to the Allen ISH and reference atlas to determine the largest region with pia-to-white matter sampling in each dataset. The final segmented and filtered data (summarized in Table 1) were used as the input datasets for downstream cell type assignment analysis.

### Comparison of gene properties across experimental protocols

**Figure 2:**
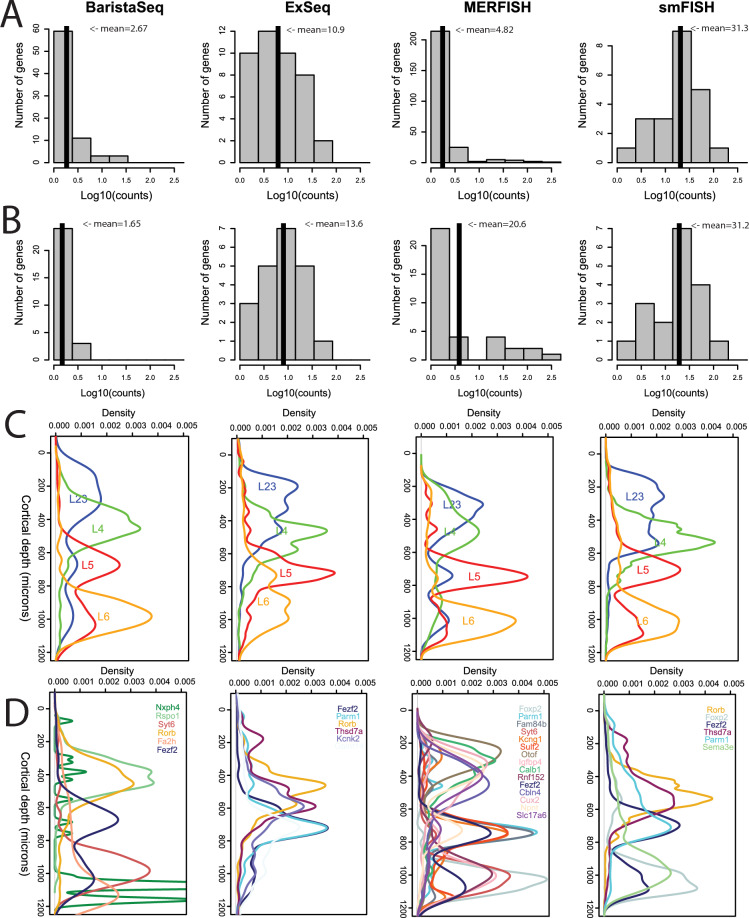
Comparison of gene properties across experimental protocols. (**A**) Distribution of average number of reads in all cells with at least one read for each gene in the experiment. Vertical lines represent the average value in the histogram. (**B**) Distribution of average number of reads in all cells with at least one read for the subset of genes in the experiment found in at least two other experiments (up to 40 total). Vertical lines represent the average value in the histogram. (**C**) Density plot of spots across the axis perpendicular to cortical layers (y-axes) for sets of genes marking L2/3 (*Cux2*, *Lamp5*, *Cxcl14*), L4 (*Rorb*, *Rspo1*), L5 (*Fezf2*, *Parm1*), and L6 (*Sema3e*, *Foxp2*, *Syt6*) in mouse VISp. At least one gene from each layer list was assayed in each experiment (Supplemental Table S1). Individual genes are pooled to generate a single density curve per layer. Densities (x-axes) are shown in the same scale across all panels in (**C, D**). (**D**) Density plot of up to the 15 genes with the highest maximum density and with maximum density ≥ 0.0025. Genes are color-coded as shown.

The spatial methods use different reagents, tissue processing steps, barcoding approaches, and amplification methods, resulting in a very different number of genes included in each experiment (Table 1) and different requirements for which genes can be successfully probed. With these constraints in mind, gene sets were selected to overlap across experiments to the extent possible to allow comparison between studies. We found that while smFISH and ExSeq had relatively fewer genes per experiment (Table 1), the average number of transcript molecules detected per cell tended to be higher than for MERFISH or BaristaSeq (Fig. 2A), even when considering only common genes among experiments (Fig. 2B).

Glutamatergic (and to a lesser extent GABAergic) neurons show strong laminar patterning in mouse VISp, and many genes have been well described as showing layer-restricted expression^34^, providing a useful ground truth for assessing the accuracy of detecting expression of a subset of genes in each experiment. For all experimental protocols, at least one gene marking L2/3 (*Cux2*, *Lamp5*, *Cxcl14*), L4 (*Rorb*, *Rspo1*), L5 (*Fezf2*, *Parm1*), and L6 (*Sema3e*, *Foxp2*, *Syt6*) in mouse VISp were assayed; in all cases these genes showed maximal expression at the expected cortical depth (Fig. 2C). Additional computationally-derived genes included in the assays showed layer restriction in MERFISH, and to a lesser extent the other experimental protocols (Fig. 2D). Together, these results suggest that sufficient information exists from the included gene panels to assign segmented cells to reference cell types at the level of major inhibitory and excitatory subclasses.

### Cell-based cell type matching

#### Individual matchings

Six cell type matching algorithms (ATLAS^26^, FR-Match^27,28^, map.cells*^4^, mfishtools^29^, pciSeq^30^, and Tangram^31^) were applied to assign reference scRNA-seq cell types to each segmented cell with an associated confidence score (or probabilistic assignment) based on the cell-by-gene count matrix (see “Methods” section). Confidence scores are metrics with values in the [0,1] range, but are defined differently in each algorithm (see “Methods” section and citations). Applying the cell type matching algorithms produces a cell-by-type matching matrix as a primary output, consisting of probabilistic assignment of each segmented cell to each of the reference cell types at the subclass level. Deterministic cell type assignment for each spatial cell is defined as the reference cell type that has the highest confidence score for each algorithm.

In this subsection and the next, we focus the discussion of cell type matching performance on MERFISH data; similar analyses were applied to the other spatial data as well. Similar but less refined matching results were observed, potentially due to the fact that fewer genes were probed in those gene panels. For further exploration, all spatial data on VISp are publicly available as a data resource at https://spacetx.github.io/data.html.

**Figure 3:**
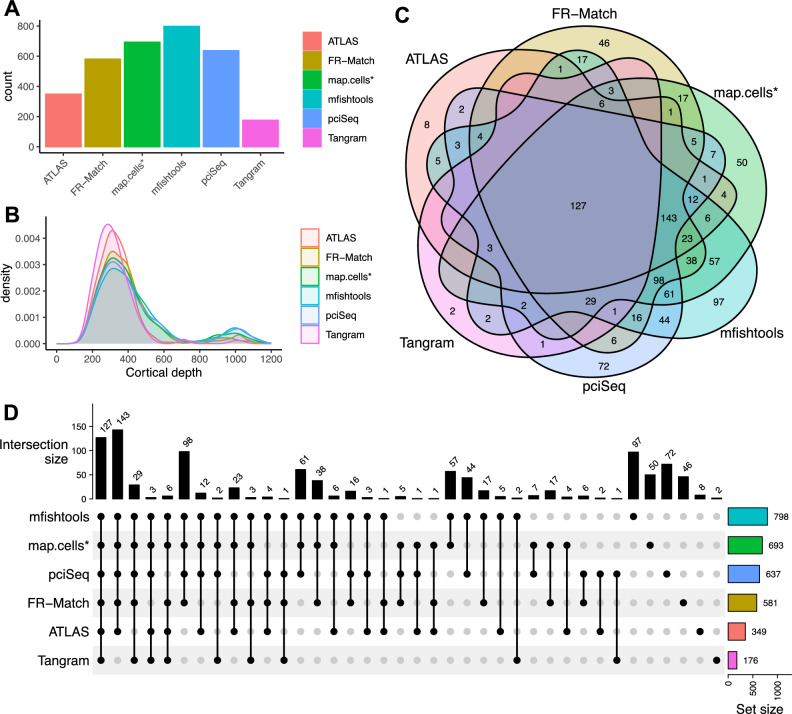
Cell type matching performance comparison on the L2/3 IT subclass of MERFISH data. Six computational methods were applied to match/assign reference cell types to the spatial cells. (**A**) Number of cells matched to the L2/3 IT subclass by each individual method. (**B**) Spatial distribution of the cells matched to the L2/3 IT subclass by each individual method. X-axis is the spatial axis perpendicular to cortical layers measured as distance (μm) from pia (left end: upper layer, right end: deeper layer). (**C**) Overlapping of cells matched to the L2/3 IT subclass by each individual method. (**D**) Breakdown of the intersections of cells matched to the L2/3 IT subclass by individual methods.

A key challenge for the deterministic assignment of cell types was the extensive differences observed among the individual matching results without the availability of a gold standard result to compare against, although some information about expected spatial distributions is available based on scRNA-seq data^4^. For example, the deterministic cell type assignment for the L2/3 IT subclass showed substantial differences in the number of matched cells (Fig. 3A) and the spatial distribution of the cells matched to the same subclass (Fig. 3B) among the individual matching methods. The differences were also reflected in the substantial amount of disagreements of cells matched to the same subclass (Fig. 3C,D).

In this example, the L2/3 IT subclass is a relatively abundant cell population consisting of intratelencephalic (IT) neurons that are expected to appear in supragranular cortical layers (layers 2 and 3). Out of the 2150 MERFISH cells for cell type matching, the number of cells matched to the L2/3 IT subclass were 349 (ATLAS), 581 (FR-Match), 693 (map.cells*), 798 (mfishtools), 637 (pciSeq), and 176 (Tangram) in the individual matching results (Fig. 3A). In all cases, the great majority of cells matching to L2/3 IT are found in supragranular cortex, as expected, although the exact footprint of this layer and number of off-target matchings varied by method (Fig. 3B). All six methods identified 127 cells in common (Fig. 3C). Tangram matched the smallest number of cells to the L2/3 IT subclass, followed by ATLAS. Most of ATLAS and Tangram matched cells were matched equivalently by the other methods, which may suggest that these methods have higher precision (a.k.a. positive predictive value) but lower sensitivity for this subclass. The other four methods identified 397 L2/3 IT cells in common (Fig. 3D), suggesting there is a relatively abundant L2/3 IT cell population (18% of all segmented cells) identifiable by the majority of methods. We may regard the method-specific cells (8 for ATLAS, 46 for FR-Match, 50 for map.cells*, 97 for mfishtools, 72 for pciSea, and 2 for Tangram) as cells that may have weaker signal and more noise in their combinatorial marker gene expression pattern; these noisy cells appear to be the major source of the observed spillover effect in the layer distributions (Supplementary Fig. S1) for this specific cell subclass.

Spatial coordinate plots with confidence score intensities for each individual matching are available in Supplementary Figs. S2–S7. The highest confidence score as the deterministic cell type assignment for each cell are plotted. All methods were able to recapitulate the laminar pattern of neuronal cells to some extent, particularly with respect to cells matched with higher confidence. Because these precision vs. sensitivity results for L2/3 IT are not necessarily representative of results from other subclasses, and since we do not have a ground truth result to assess performance against, we are not able to conclude that any specific method outperforms the others on cell type matching. Instead, we chose to computationally combine these matching methods, as has been done previously for other computational tasks such as cell type clustering^35^ and cell morphology tracing^36^.

#### Combined matchings

It is likely that the individual cell type matching methods have different advantages and experimental biases, and often produce different cell type assignments, especially in those cells with fewer total transcripts or less confident segmentation boundaries (Supplementary Fig. S8). Assuming that the majority of individual methods would produce some level of accurate cell type matching/assignment, combining their results using an ensemble approach may provide the best classification result. We used two different strategies to combine all individual matching results in the ensemble meta-analysis—GMCS and NWCS, each producing a re-calculated confidence score matrix for determining the consensus cell type assignment. The GMCS combined matching approach considers each individual matching result as the vertex of a polygon whose geometric median, the point with minimum average Euclidean distance from these vertices, serves as the combined result (see “Methods” section). The NWCS combined matching approach is a rank-based weighted average of the confidence scores from each individual matching method using only the highest score for each cell (see “Methods” section). For all matching results, deterministic cell type assignment is defined as the cell type with the highest confidence score for a given cell. The confidence scores could be used as a quantitative metric of matching strength. However, they are defined differently and show very different distributional properties from each matching method (Supplementary Fig. S9). Even though all confidence scores are in the range of [0,1], they are therefore not directly comparable across individual matching results. As such, the ranks (i.e., ordered statistics) of the scores are pragmatically more useful, with deterministic cell type assignment using the top-ranked confidence score.

**Figure 4:**
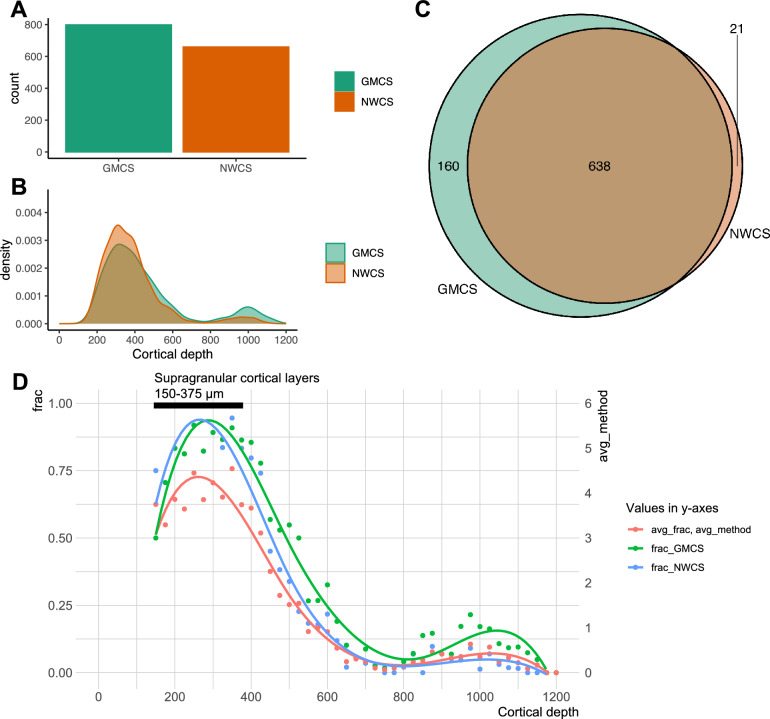
Combined cell type matching performance on the L2/3 IT subclass of MERFISH data. Two ensemble approaches were applied to combine the individual matching results, resulting in two combined matchings—GMCS and NWCS. (**A**) Number of cells matched to the L2/3 IT subclass in each combined matching result. (**B**) Spatial distribution of the cells matched to the L2/3 IT subclass in each combined matching result. X-axis is the spatial axis perpendicular to cortical layers measured as distance (μm) from pia (left end: upper layer, right end: deeper layer). (**C**) Overlapping of cells matched to the L2/3 IT subclass in the combined matching results. (**D**) Comparison between averaged summary statistics of the individual matching results and summary statistics of the combined matching results. Each dot represents a bin size of 25 μm in the cortical depth axis. Avg_frac is the fraction of cells matched to the L2/3 IT subclass in the cortical depth bin averaged over individual matching methods. Avg_method is the average number of individual methods that matched a cell to L2/3 IT subclass in the cortical depth bin. Frac_GMCS and Frac_NSCS are the fraction of cells matched to the L2/3 IT subclass in the cortical depth bin for the combined matching results. Supragranular cortical layers are indicated by the black bar.

Using the L2/3 IT subclass as an example, the combined matching results are more similar in the number of cells (798 for GMCS and 659 for NWCS) matched to the subclass (Fig. 4A) and the spatial distributions mostly aligned (Fig. 4B). Between the two combined matching results, the vast majority of the cells matched to the same subclass (Fig. 4C), indicating strong agreement between the two combined matchings. The combined matching GMCS and NWCS assigned 31% and 37% of all MERFISH cells to the L2/3 IT subclass, respectively, although there is still some spillover of the matched cells in the layer distribution. Using the ensemble approaches, more cells in the expected supragranular cortical layers are matched to the L2/3 IT subclass compared to individual matchings (Fig. 4D). On average (arithmetic mean), up to 75% (red curve) of cells in the supragranular cortical layers are matched to L2/3 IT across all 6 individual methods. In contrast, up to 90% (green and blue curves) of cells in the supragranular cortical layers are matched to this subclass using these ensemble approaches, suggesting that these ensemble methods are effective producing a highly compacted consensus result, whereas simple combinations (e.g., arithmetic mean) of the individual matching results are not effectively aggregating the cells matched to L2/3 IT subclass towards the expected cortical layers.

Considering all cells, the two combined matching results produced cell type assignments with 83% (= number of cells assigned to the same subclass/total number of cells) of cells being assigned to the same subclass, overcoming the large differences among individual matching results. The combined confidence score intensity matching plots for all cells are available in Supplementary Figs. S10–S11; and the distribution of all cells in cortical layers by each combined matching are in Supplementary Fig. S12. Though the distributions of matched cells in cortical layers are very similar for the abundant GABAergic and glutamatergic subclasses between the two combined matchings (Supplementary Fig. S12), they differ in rare and non-neuronal subclasses (e.g., Meis2, Endothelial, and Macrophage), suggesting that it is more difficult to detect and match rare cell types in spatial transcriptomics. Overall, these results suggest that, while individual matching algorithms may have different strengths and biases leading to somewhat different results, the ensemble methods provide a more robust cell type matching/assignment for the spatial cells.

#### Laminar distributions of neurons from computational methods

**Figure 5:**
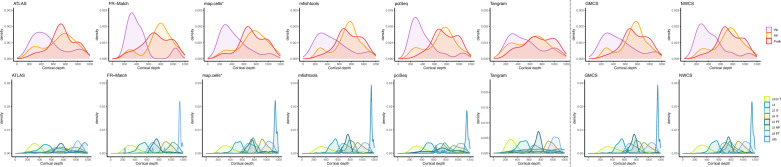
Spatial distributions of inhibitory and excitatory neurons in individual and combined matching results of MERFISH data. Top: distributions (cortical depth) of inhibitory neurons of Vip, Sst, and Pvalb types show a peak of Vip type in upper layers and peaks of Sst and Pvalb types in deeper layers. Bottom: distributions of excitatory neurons follow the major laminar pattern expected, with various minor peaks in different methods. For detailed view, see the corresponding spatial coordinate plots of the matched cells for each subclass using each method in Supplementary Figs. S2–S7 and S10–S11.

The spatial distribution of many known neuron types have been studied through frozen dissection, RNA scope staining, confocal imaging, etc. For example, Fig. 1 in Tasic et al.^4^ shows the laminar patterns of mouse VISp reference scRNA-seq cell types in layer dissections. With the MERFISH data and all computational matching results, we plot the calculated spatial distributions of the matched inhibitory and excitatory neurons in Fig. 5. For the inhibitory neuron types, the Vip neurons are distributed in upper layers and the Sst and Pvalb neurons are distributed in deeper layers as expected. For the excitatory neuron types, in general, the major peak of each spatial distribution curve is found in the expected layer, but with varying width of the major peak and minor peaks in some cases.

#### Individual matching method agreement across protocols

Experimental protocols showed different detection sensitivities across platforms (Table 1 in the SpaceTx Consortium paper^19^), thus individual matching method performance also varied across protocols. Since there is no ground truth, we use the empirical evidence of pairwise agreement (Cohen’s Kappa^37^) between individual matching results as a metric to quantify the consistency of the cell type matching results for each protocol. Table 2 shows the best Kappa value (higher = better) and the most agreed upon pair of methods for each protocol. Kappa values of MERFISH, smFISH and ExSeq indicate substantial agreement between the best pair, all of which include mfishtools. Kappa value of BaristaSeq data shows modest agreement, indicating there is no good cell type matching result for this spatial method suggesting that more investigation of the experimental protocol may be needed. Table 2 suggests that mfishtools may be the best first choice of cell type matching for the other three protocols. All pairwise Kappa values are in Supplementary Table S2

**Table 2:** Best pairwise agreement (Cohen’s Kappa) between individual matching methods for each protocol.

Protocol	Method 1	Method 2	Kappa
MERFISH	map.cells*	mfishtools	0.70
smFISH	FR-Match	mfishtools	0.84
BaristaSeq	Tangram	ATLAS	0.23
ExSeq	mfishtools	pciSeq	0.75

See full table in Supplementary Table S2. Kappa values: ≤ 0 indicates no agreement, 0.01–0.20 indicates slight agreement, 0.21–0.40 fair agreement, 0.41–0.60 moderate agreement, 0.61–0.80 substantial agreement, and 0.81–1.00 almost perfect agreement^37^.

### Segmentation-free cell type matching

**Figure 6:**
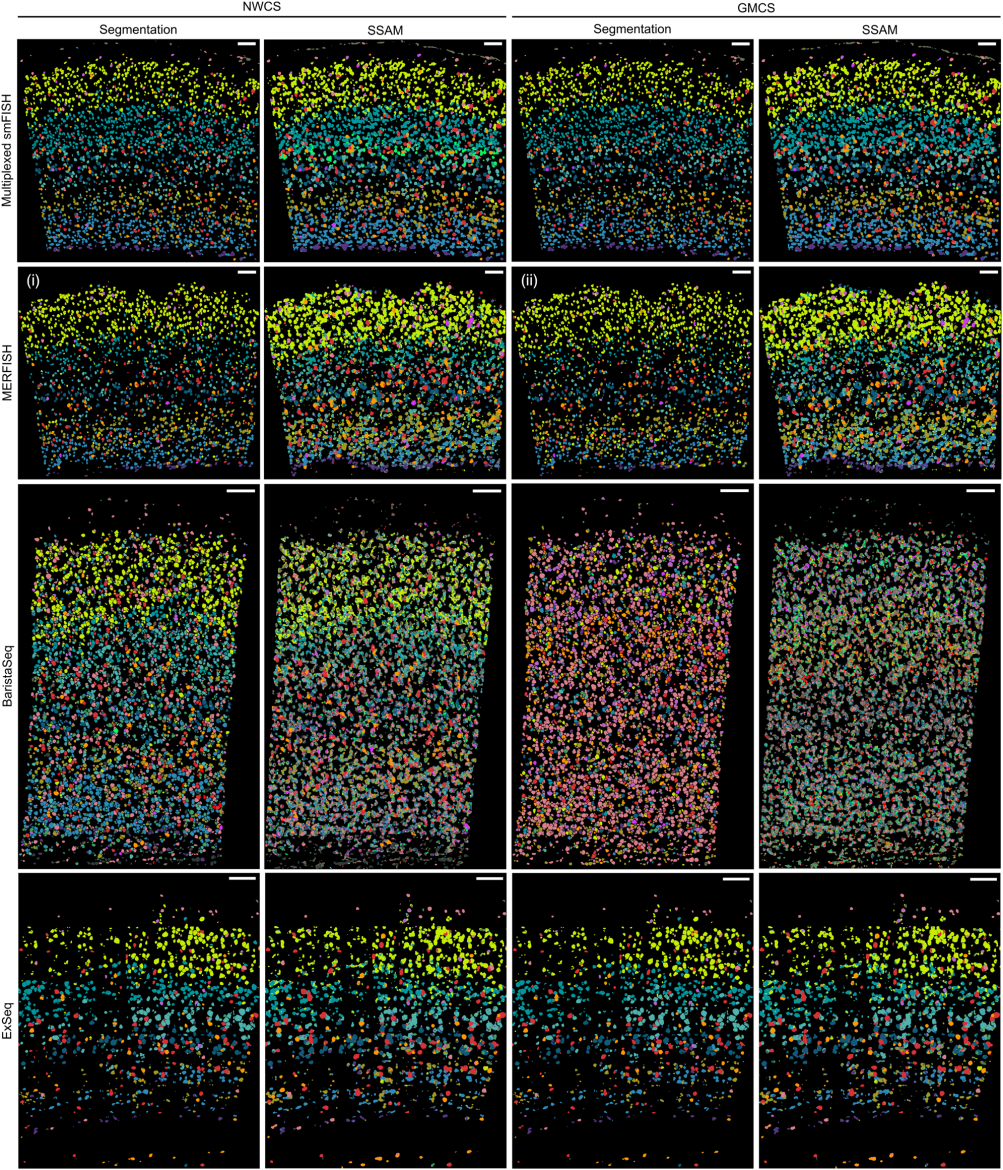
Side-by-side comparison between the segmentation and the SSAM results. Images of VISp tissue from four experimental methods (rows) with either cells (segmentation columns) or spots (SSAM columns) color coded by cell type assignments based on the NWCS (left columns) or GWCS (right columns) combining methods. The colors of each cell type can be found in Fig. 1. The scale bars represent 100 μm in all panels. Panels show similar laminar patterns in each row with some exceptions noted in the main text. Subplots (i) and (ii) correspond to the distributions in Fig. 4B, where the L2/3 cells (bright green color) distribute in a slightly wider top layer and have more occurrences in deeper layer in the GMCS subplot (ii) compared to the NWCS subplot (i).

Working directly on the spot-by-gene matrices, the SSAM^32^ framework was used to perform and visualize segmentation-free spatial cell type assignments. SSAM performs pixel-wise cell assignment based on spatial distribution of each gene’s expression pattern and does not require prior cell segmentation. We used SSAM as an alternative to generate spot-based cell type matching results and as a computational validation of the ensemble meta-analysis results on the segmented cells. Here, SSAM guided-mode was used to create cell type assignments, guided by the mean log-normalized gene expression of the combined cell type matching results (GMCS and NWCS) (Supplementary Figs. S13–S20). In general, the resulting SSAM cell type assignments and the segmented cells with cell type assignments from combined matching results showed visual similarity in both meta-analysis combining strategies for all spatial experimental methods (Fig. 6). One exception was the GMCS-based cell type assignment of BaristaSeq. This was due to the low quality of the consensus matching; both the segmentation-based and the SSAM results did not match the previously known layer structure of the visual cortex.

However, detailed comparison revealed that there are unique spot-based cell type assignments determined by SSAM that were not found in the segmentation-based approach, e.g., the VLMC subclass in the multiplexed smFISH dataset (olive-colored cells in Supplementary Fig. S21A). The marker gene of the VLMC subclass *Alcam* showed a very similar gene expression pattern as SSAM identified (Supplementary Fig. S21B), which strongly assumes the existence of cells in the region. This observation demonstrates that SSAM could be used as an alternative method to quickly visualize spatial cell types that were possibly missed by prior cell segmentation. Also, due to the lower density of mRNAs captured by BaristaSeq compared to other FISH methods, the SSAM cell type assignment of BaristaSeq was noisy. Overall, this analysis shows that a spot-based approach, such as SSAM, is equally capable of revealing good-quality segmentation-free cell type assignment for spatial transcriptomics pixel data, especially using the guided mode analysis when precise gene signatures are given.

Another interesting difference between the segmentation and segmentation-free approaches is that, in the smFISH results, SSAM introduced a spatial pattern of a thin layer of spots in deep layer 4 (bright green cells in Supplementary Fig. S21C) guided by one segmented cell that was not assigned as L4 in the spatial neighborhood. The segmented cell was assigned to the CR subclass (Supplementary Fig. S21A, right panel), a rare and transient class of neurons found in mammalian cortex. Without knowing the ground truth, the assignment of the CR cell could not be validated using the limited number of probe genes in the smFISH panel (22 genes). However, the subtle difference between this cell and the L4 cells were captured from the meta-analysis of the cell type matching results, based on which, SSAM further captured a series of spots that show this similar yet distinct expression profile (Supplementary Fig. S21D,E). As previously reported, this series of inferred cell spots might be a cell state split from the L4 cells, as suggested in^32^. All these observations suggest that segmentation-free approach can provide complementary insights based on the spot data directly.

### Cytosplore viewer for comparative visualization of spatial protocols

#### ScRNA-seq based gene imputation for data visualization

**Figure 7:**
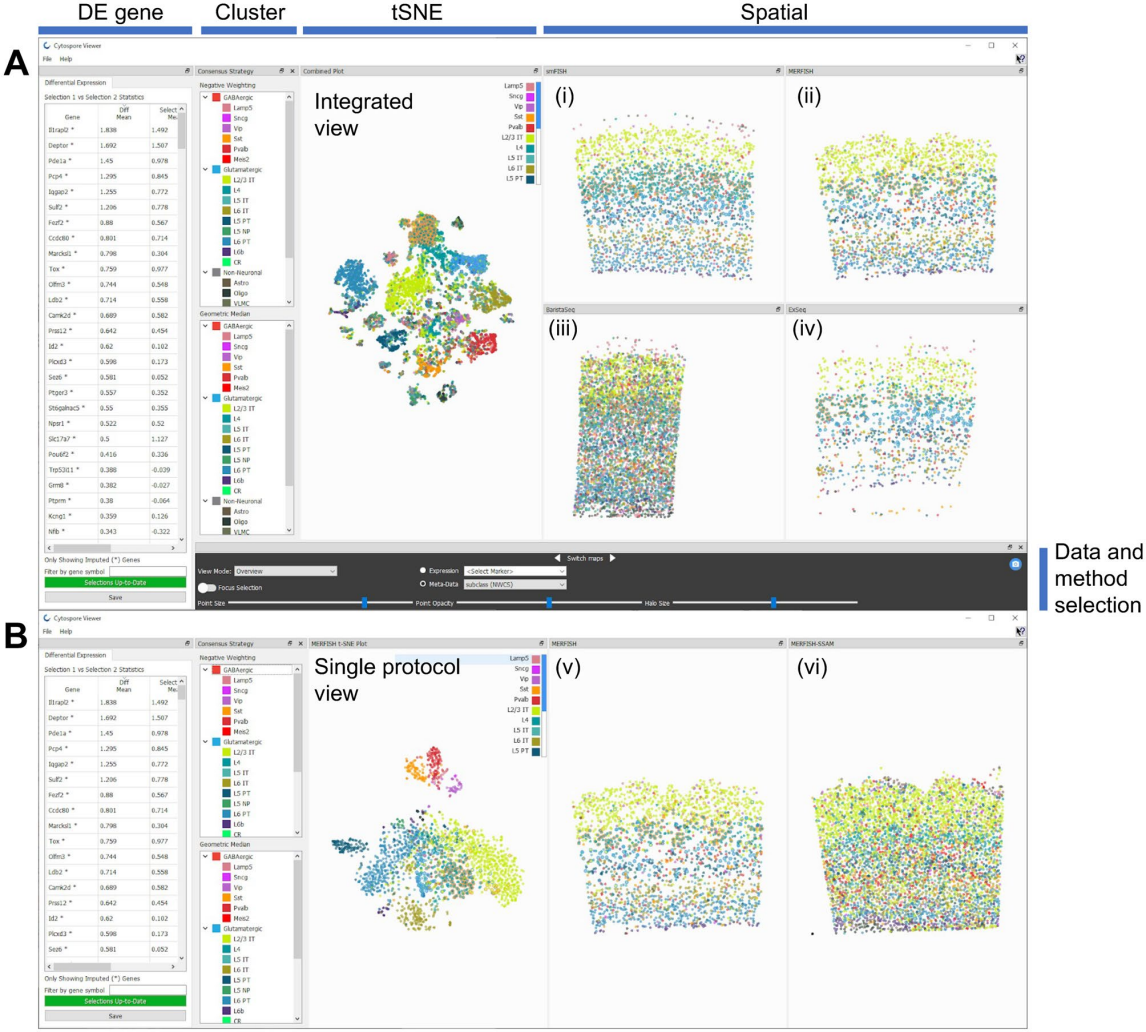
Cytosplore Viewer enables comparative visualization of the SpaceTx data and methods, enabling cell selection from cluster taxonomies (cluster panel), tSNE of single cells based on expression profiles (tSNE panel) and spatial coordinates of cells/local maxima (spatial panels). (**A**) Cross-protocol comparison view: an integrated tSNE map of all cells enables side-by-side comparison of spatial patterning of both consensus matching results on smFish (i), MERFISH (ii), BaristaSeq (iii) and ExSeq (iv), as well as differential expression analysis of cell selections (DE gene panel). (**B**) Single protocol comparison view enables comparing the consensus matchings in the segmentation-based methods (v) and segmentation-free SSAM results (vi) for the individual spatial protocols. Viewing panels are highlighted on the top; data and method selection panel is highlighted to the right of the figure. The NWCS results are shown in both (**A, B**); MERFISH data and results are shown in (**B**). Data and methods can be selected in the data and method selection panel.

To compare the different spatial transcriptomics protocols, a combined embedding was generated. A major challenge is that each protocol measures a different set of genes, and the number of shared genes is very small. The four protocols (MERFISH, smFISH, BaristaSeq, and ExSeq) share only six common genes, while the number of genes measured per dataset varies from 22 to 253 genes; the union of these gene sets contains 314 genes (Supplementary Table S1). To address this problem, we applied SpaGE^38^ to impute the expression of the missing genes in each dataset separately and obtain a total of 314 genes per dataset. For each spatial dataset, SpaGE integrates the spatial data with a reference scRNA-seq dataset measured from the same tissue and provides prediction for the expression of the missing genes. For example, the MERFISH dataset has 253 measured genes, SpaGE was applied to impute the expression of the 61 remaining genes. Additionally, to reduce batch effects in the joint multi-protocol embedding, we used SpaGE to re-impute the expression of the measured genes in each spatial dataset separately, using a leave-one-gene-out scheme. Taking the MERFISH dataset as an example, SpaGE uses 252 genes for integration with the reference scRNA-seq dataset and provides predicted expression, for the left-out gene, imputed from the scRNA-seq data. This process is applied for all measured genes in each spatial datasets to ensure that all spatial datasets are aligned to the reference scRNA-seq data, and that the expression of all genes is obtained from the same (scRNA-seq) domain. Finally, we generated a combined tSNE embedding for all four spatial datasets using the imputed expression matrix of all 314 genes (Fig. 7A tSNE panel).

#### Comparative visualization between protocols

For comparative visualization of the different spatial protocols, the Cytosplore Viewer (https://viewer.cytosplore.org) was extended with functionality for side-by-side visualization of multiple spatial datasets, in combination with the consensus clustering described above. A comparative view was developed that enables interactive selection from the consensus clustering hierarchy (Fig. 7A), or from the joint tSNE embedding combining cells from all spatial protocols. Also, either the measured or imputed expression values can be painted on the spatial and tSNE maps enabling comparison of spatial expression patterns across spatial protocols. Finally, functionality for differential expression (DE) analysis between two manual selections (drawn either in the spatial maps or the tSNE plots) was implemented, enabling quick retrieval of differentially expressed genes between regions or cell types. Comparing cell selections within one protocol returns DE of the measured genes for that protocol. Manual selection of cells in the combined tSNE map returns DE of the imputed gene sets.

#### Single protocol and SSAM visualization

Apart from the comparative visualization, functionality for visualizing individual protocols was developed, consisting of a linked spatial and tSNE map (Fig. 7B). The tSNE maps were computed on the measured cell-by-gene expression matrices of the individual spatial datasets. Since SSAM omits the step of direct cell segmentation, estimating local correlation between SSAM Kernel Density Estimate profiles and the cluster prototypes, direct comparison between cell-segmented and estimated local correlation maxima was not possible. As such, the individual SSAM local maxima-by-gene matrices were included in the single protocol visualizations.

## Discussion

Spatial transcriptomics methods are fast evolving, which requires up-to-speed development of data analysis pipelines. Significant emphasis has been devoted to computational algorithms focused on the segmentation step of the imaging-based spatial transcriptomics analysis pipeline^25,39–41^. However, limited focus has been placed on investigating the performance of spatial cell type matching in downstream analyses. This study evaluates the scRNA-seq-reference-based cell type matching performance across spatial transcriptomics experimental methods and cell type matching computational methods on systematic benchmark datasets generated on serial tissue sections with soma proximity.

In the SpaceTx project, the primary use for the reference taxonomy is to define a standard set of cell types and gene expression profiles for (1) defining marker gene panels and (2) mapping cells from spatial data sets. While there are no explicit requirements for reference scRNA-seq or the query spatial transcriptomics data sets; however, larger data sets (with more cells, reads per cell, and genes per study) will generally lead to higher confidence mapping. Given the improvements in technology since the start of the SpaceTx project, essentially every experiment generated in the past few years has sufficient size. Finally, while re-clustering the reference to a higher resolution could have been helpful in principle, we would recommend using reported cell type definitions from reference data sets as is for better cross-study comparison.

A particularly crucial step for cell type matching is data normalization for cross-platform or cross-study matching. It is well-observed that gene expression counts from in situ sequencing for spatial transcriptomics and scRNA-seq show very different data distributional properties, including maximum counts, median counts per gene, zero-inflation effect, etc. However, there is no one-size-fits-all solution to address the data normalization challenge. Different data normalization techniques (e.g., min–max scaling, z-score transformation, quantile alignment, etc.) may be required for different computational methods. In this project, uniformly segmented cell-by-gene count matrices were distributed to each team that participated in the SpaceJam Hackathon. Data normalization for different spatial experiment protocols and the reference scRNA-seq data was performed by each team based on their own experience and judgment. Though data normalization evaluation is not the focus of this manuscript, we acknowledge that some differences in the results may come from biases originating from the method-dependent normalization steps. Some of the computational methods considered in this manuscript reported their own evaluation of the normalization step on the method performance. For example, FR-Match has reported a thorough evaluation of the normalization step for the cross-platform and cross-study cell type matching problems, including matching spatial and single-cell transcriptomics data^28^. A recent publication also reported a systematic evaluation of the theoretical and practical aspects of data transformations and normalizations for single-cell RNA-seq data^42^.

In this manuscript, we first compared gene detection sensitivity and gene expression patterning across spatial experimental methods, which revealed high variability and very different dynamic ranges in the in situ hybridization data across different experimental protocols. We also presented a systematic evaluation of the individual cell type matching algorithms and the combined matching strategies using the MERFISH dataset as an example. The cell-based cell type matching algorithms were applied following the same segmentation step on the image data. Individual matching results varied largely in their metrics of matching confidence as well as their deterministic cell type assignments, among which no overall “winner” could be claimed without a gold standard result to compare against. Given the variable performance of individual matching results, we used ensemble meta-analysis approaches to combine these individual matchings to form consensus results. The meta-analysis approaches largely improved the agreement between the consensus matchings, where the majority of the cells have the same cell type assignment by the two combined matching strategies. One exception is the NWCS and GMCS results for BaristaSeq, which may further suggest that rank-based approaches (e.g., NWCS) can be practically more useful for meta-analysis and more robust to the variations from individual matching results. Using the spot-based cell type matching algorithm, similar results as the consensus results could be efficiently obtained without explicit segmentation, given that precise gene signatures are available.

A Cytosplore Viewer compilation allows all spatial cells from all evaluated experimental protocols to be viewed in an integrated tSNE map based on the SpaGE-imputed expression scores from scRNA-seq reference data. This enables interactive selection of cells (either through free-form selection or per cell type subclasses), confirming the consistency of the layer patterns across spatial protocols. Differential analysis between free-form cell selections proved particularly useful for identifying gene expression gradients across cortical layers and confirming them across protocols. A side-by-side comparison between the segmentation-based workflow and segmentation-free SSAM revealed a larger density of local maxima detected by SSAM compared to the segmentation-based analysis. However, the spatial patterning of cell type subclasses was highly conserved between both methods. Finally, a direct comparison between both combining strategies revealed similar cell type matching results for smFISH, MERFISH and ExSeq. For BaristaSeq, the combined matching by GMCS resulted in inconclusive results, whereas the NWCS matching still performed reasonably well.

Alongside individual publications utilizing SpaceTx tissue and data^12,28,43^, the SpaceTx project produced three consortium-level outputs: (i) a summary manuscript of the overarching SpaceTx Consortium effort^19^, (ii) this manuscript focusing on the computational benchmarking and meta-analysis, and (iii) a companion website (https://spacetx.github.io/) for data and results dissemination. This manuscript presents both accomplishments and lessons learned from the SpaceJam Hackathon. Brain tissue was chosen for this evaluation because neuronal cell types present unprecedented granularity, e.g., 116 cell types in the mouse VISp reference scRNA-seq data. Though our analysis showed the spatial transcriptomics protocols evaluated in this project were not able to capture the cell type difference at this granularity, a more recent MERFISH study with 4000 probe genes revealed more than 100 spatially-resolved cell types in human middle temporal gyrus^44^. The computational benchmarking framework and the meta-analysis approaches presented in this study could be directly applied to these newly generated data. Our results of performance benchmarking at the 24 subclass types of mouse VISp are expected to be generalizable to datasets of other tissue or organ system that presents similar cell type granularity. At the time of the Hackathon, though no single computational method could provide a complete solution to all the challenges in the spatial transcriptomics data, the post-Hackathon meta-analysis revealed that improvement could be achieved by combining multiple methods using ensemble approaches. More recently,^45^ reports the benchmarking performance of 16 integration methods for spatial and single-cell transcriptomics data and suggests Tangram is one of the top methods. The latest version of the single cell integration method Seurat v4^46^ also extends its pipeline to integrate 10 × Visium and Slide-seq spatial transcriptomic data with scRNA-seq reference. We also considered these technologies in the SpaceTx project, and more details and results can be found in the publicly available consortium-level outputs. Our study together with others provide a comprehensive landscape of the field.

The spatial transcriptomics community is growing rapidly with advancements in both experimental and computational methods. These advancements have provided valuable insights into the molecular architecture of the brain, including neuronal cell type classification, spatial pattern recognition, spatially differential gene expression, etc. The scope of this manuscript focused on the cell type classification of spatial transcriptomics data using the reference cell type transcriptional profiles from an scRNA-seq atlas. Applications to spatial pattern recognition and spatially differential gene expression are also important topics but beyond the scope of this manuscript. It will be interesting to apply the meta-analysis framework proposed here to benchmark the performance in other applications in the future. For downstream cell type analysis, challenges and opportunities co-exist as benchmarked analysis pipelines are lacking. A major goal of future work is to promote standardization in data formats and computational methods, including methods for marker selection, probe design, cell segmentation, cell nuclei and boundary delineation, cell type matching, and spatial pattern recognition. It will need the community to provide public access to large, high-quality, uniformly collected datasets from all current spatial transcriptomics methods, in common standard file formats, to accelerate innovation in the computational analysis of such data.

## Methods

### Cell-based cell type matching algorithms

We evaluated six computational cell type matching algorithms, namely ATLAS^26^, FR-Match^27,28^, map.cells*^4^, mfishtools^29^, pciSeq^30^, and Tangram^31^.

ATLAS (A Tool for Learning from Atlas-scale Single-cell multi-omic measurements) (https://github.com/spacetx-spacejam/edv) uses a neural network classifier that applies a central moment discrepancy (CMD)^47,48^ term as a domain regularizer to map cell types discovered in the scRNA-seq data onto the spatial data. The input to ATLAS is the scRNA-seq measurements and the corresponding cell type labels in addition to the spatial transcriptomic measurements at single cell resolution. Using these inputs, ATLAS maps the cell types discovered from the scRNA-seq data onto each cell in the spatial transcriptomics data.

The FR-Match algorithm^27,28^ (https://github.com/JCVenterInstitute/FRmatch) requires an initial de novo clustering of the spatial transcriptomics data, which provides a supervised mode for the algorithm. Both the candidate spatial cell clusters and the scRNA-seq reference cell types were input to the algorithm, and the best-matched reference cell types for each spatial cell were obtained using the cell-to-cluster function (FRmatch_cell2cluster) implemented in the “FR-Match” R package.

The map.cells* algorithm uses a derivative from the map.cells function in “scratch.hicat” R package^4^ (https://github.com/AllenInstitute/scrattch.hicat) that was altered to make it more suitable for smaller gene panels. It is a bootstrap-based method that uses Pearson correlation to assess the similarities between cells and cell type clusters.

The mfishtools algorithm^29^ (https://github.com/AllenInstitute/mfishtools) also uses Pearson correlation to match cells from spatial transcriptomics method to cell type cluster medians in a scRNA-seq reference dataset. This algorithm first applies filtering and scaling strategies to the mFISH and scRNA-seq datasets, and then uses correlation-based assessment to find the best fitting cell type cluster. There are several parameters allowing flexibility in filtering and analysis. Probabilities for cell type assignment were approximated using the following pseudocode:

$$\begin{aligned} &\texttt{scaledCorrelation = pmax(y-(max(y)/2),0)} \wedge \texttt{2}\\ & \texttt{probability = scaledCorrelation/sum(scaledCorrelation)} \end{aligned}$$

where y is the vector of correlations between a given spatial transcriptomics cell and the median expression of each scRNA-seq cell type cluster. Finally, several functions for visualization of matching results and assessment of matching accuracy are included in the mfishtools R package and were applied in this study. A vignette for application of this method is available as part of the “mfishtools” R library.

Probabilistic Cell typing by In situ Sequencing (pciSeq)^30^ (https://github.com/acycliq/pciSeq) is a Python package for probabilistic cell typing by in situ sequencing. It uses a Bayesian algorithm, leveraging scRNA-seq data to first estimate the probability of each spot belonging to a cell and then each cell to a scRNA-seq cluster. Spots dataframe, segmentation image labels, and scRNA-seq data are required inputs to the algorithm.

Tangram^31^ (https://github.com/broadinstitute/Tangram) is distributed as a Python package, based on PyTorch and scanpy. Tangram requires as input a single-cell (or single-nucleus) gene expression dataset and a spatial gene expression dataset. Tangram learns an alignment for the single-cell data onto space by fitting gene expression on the shared genes. The output of the matching algorithm is a cell-by-spot matrix, that gives the probability for cell $i$ to be in spot $j$. Using this matching matrix, Tangram can project any annotation (e.g., cell types) from single-cell data onto space. The standard pipeline (with cell-level mapping) has been applied, using functions tg.map_cells_to_space for learning the matching and tg.project_cell_annotations for projecting cell types computed on scRNA-seq data onto space.

### Combining strategies for consensus matching

#### Geometric Median Combining Strategy (GMCS)

Given the above combining strategy weighing certain matchings over others, we also introduce an independently-developed combining strategy using a geometric median approach that considers each matching equally. Given $m$ matchings, each matching $c$ cells to a probability distribution over $n$ potential cell types, we create a $m$-gon (polygon with $m$ vertices) with vertices in the $n$-dimensional space (${R}^{n}$). For each of these polygons, we then find the geometric median, i.e., the point $p\in {R}^{n}$ at which the sum of the ${L}_{2}$ norms from $p$ to each vertex in the polygon is minimized. Intuitively, such a point considers each of the individual matchings equally, as having a point $p$ closer to one individual matching's vertex than another would not minimize the sum of the ${L}_{2}$ norms. The confidence with which this matching assigns cell types is consequently a function of how similar or disparate constituent matchings are. Accordingly, certain data modalities for which the individual matching results largely disagree with one another, e.g., BaristaSeq, resulted in not-as-well-classified cells, whereas data modalities in which each cell's corresponding polygon is of relatively small area, e.g., MERFISH, yielded very well-defined consensus matching (**Results**).

#### Negative Weighting Combining Strategy (NWCS)

A weighting approach was designed to combine the six individual cell type matching results. An evaluation of the individual matching results revealed that: (1) The probabilistic assignments (a.k.a. confidence scores) that reflect the confidence of matching for each spatial cell to each reference cell type showed very different distributions from method to method; some were more binary as either 0 or 1 and others showed more plateau distributions (Supplementary Fig. 9). (2) Despite the distributional difference, some cells were assigned to the same cell type with the highest confidence score by all of the methods (i.e., well-matched cells), whereas other cells were only matched to a cell type with a high score by only one method (i.e., inconsistently-matched cells). In order to avoid the bias introduced by the accidental assignment of those inconsistently-matched cells, we designed a negative weighting scheme to borrow the best-matched confidence score among all methods. NWCS performs the following steps to combine the individual matching results: (1) Find the best-matched cell types of each cell by keeping the cell-wise highest confidence score. (2) Assign a negative weight (− 1) to all other cell types for each cell. (3) The combined confidence score matrix is the sum of all negatively weighted confidence score matrices of each individual method. (4) The NWCS cell type deterministic assignment is the cell type with the maximum confidence score for each cell in the combined matrix.

### Segmentation-free cell type analysis method

SSAM (Spot-based Spatial cell-type Analysis by Multidimensional mRNA density estimation)^32^ analysis is a method that uses the guided mode to generate segmentation-free cell type assignments of the GMCS and NWCS consensus cell types. For all datasets (MERFISH, smFISH, BaristaSeq, and ExSeq), the kernel density estimation (KDE) was performed with the location of mRNAs of each gene with the bandwidth 2.5 μm. For SSAM analysis, the resulting vector field was normalized by a library size of 10, and then log-transformed. For GMCS and NWCS cell normalization, the mRNA count of each cell type cluster was normalized to a library size of 10 per cell, and then log-transformed. The gene expression signature of each consensus cell type was computed by taking the mean of all normalized cells in the same cluster. The resulting signatures were then mapped to the vector field, by computing Pearson’s correlations between each consensus signature to all pixels in the vector field. The resulting cell types were filtered with the minimum correlation threshold 0.6.

## Supplementary information

Supplementary Information 1.

Supplementary Information 2.

Supplementary Information 3.

## Data Availability

Experimental data collected in this study that passed quality control are available as downloadable datasets at https://spacetx.github.io/data.html. Each dataset is associated with a readme document, including contact information, cell-by-gene count matrices, mapped cell tables, and spot tables. The raw data are summarized in Table 1.

## Code availability

Data analyses were performed using open-source software. The cell segmentation method Baysor is available at https://github.com/kharchenkolab/Baysor (v0.5.0). The cell type matching algorithms: FR-Match is available at https://github.com/JCVenterInstitute/FRmatch (v2.0), map.cells* is available at https://github.com/AllenInstitute/scrattch.hicat (v1.0.0), mfishtools is available at https://github.com/AllenInstitute/mfishtools (v0.0.2), pciSeq is available at https://github.com/acycliq/pciSeq (v0.0.45), Tangram is available at https://github.com/broadinstitute/Tangram (v1.0.0). The segmentation-free algorithm SSAM is available at https://github.com/HiDiHlabs/ssam (v1.0.2). The data visualization software Cytosplore iewer is available at https://viewer.cytosplore.org (v3.0.0). The reproducible code for the results reported in this study are available at https://spacetx.github.io/results.html.
